# Term rules for simple metal clusters

**DOI:** 10.1038/srep15760

**Published:** 2015-10-26

**Authors:** Daisuke Yoshida, Hannes Raebiger

**Affiliations:** 1 Department of Physics, Yokohama National University, Yokohama, Japan; 2 Centro de Ciěncias Naturais e Humanas, Universidade Federal do ABC, Santo André, SP, Brazil

## Abstract

Hund’s term rules are only valid for isolated atoms, but have no generalization for molecules or clusters of several atoms. We present a benchmark calculation of Al_2_ and Al_3_, for which we find the high and low-spin ground states ^3^Π_*u*_ and 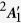, respectively. We show that the relative stabilities of all the molecular terms of Al_2_ and Al_3_ can be described by simple rules pertaining to bonding structures and symmetries, which serve as guiding principles to determine ground state terms of arbitrary multi-atom clusters.

The ground state terms (spin and angular momenta) of isolated atoms are determined by Hund’s rules^1^, which are explained by the lowering of the electronuclear attraction energy^2^,^3^,^4^,^5^. For molecules and clusters, such term rules do not exist. Group theory allows us to determine the possible ^2*S*+1^Ξ molecular terms, where *S* denotes the total spin and Ξ the symmetry species, but there is no systematic way to figure out which of these is the ground state. Direct experimental observation or quantum chemical total energy calculation is only available for a few prototype systems.

Hund’s first rule of maximum spin multiplicity holds for many organic molecules^6^,^7^,^8^, but not all^9^. Diatomic molecules on the other hand, tend to have spin singlet ground states with the exception of O_2_ and B_2_ (see e.g. refs 10, 11, 12, 13, 14, 15, 16, 17, 18, 19 for diatomic molecules of main group elements). These molecules tend to have ground states that minimize the internuclear bond length, which may be associated with a lowering of the electronuclear attraction energy, but whether or not such discussion generalizes to metallic clusters remains unknown. Moreover, recent attempts to generalize Hund’s rules for molecules^8^,^20^ or clusters^21^,^22^,^23^ only focus on a spin multiplicity rule, and trends or rules for Ξ (symmetry) terms remain completely uncharted territory.

Simple Al clusters emerge as the ideal model system to study term rules. Bulk Al is paramagnetic, but in low dimensional structures Al atoms may spontaneously align their spins. For example, strained quasi-1D chains of Al may exhibit ferromagnetism^24^,^25^, and Al_n_ clusters with even *n* = 2, 4, 6, 8 have spin-triplet ground states^21^,^26^,^27^,^28^,^29^. Al_3_ on the other hand has spin-doublet (low-spin) and spin-quadruplet (high-spin) configurations, but which one of these is the ground state remains unresolved^28^,^30^,^31^,^32^,^33^. The present benchmark study confirms that Al_2_ has the ^3^Π_*u*_ high-spin ground state and unambiguously shows that Al_3_ has the low-spin 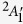 ground state. The Al_2_ high-spin state is stabilized by Fermi correlation, which is not overcome by Coulomb correlation that tends to increase the stability of low-spin terms. For Al_3_, however, the high-spin term has a symmetry broken geometry that preempts effective Coulomb correlation from taking place, thus un-stabilizing the high-spin term. Such symmetry lowering can debilitate high-spin terms of any multi-atom system. Moreover, fear each spin state, we find a simple rule for the Ξ terms. The Ξ term with least node wavefunction is most stable, and for terms with equal number of nodes, the one with most bonds is most stable. Notice that for diatomic molecules, Ξ is the angular momentum 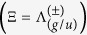 along the internuclear axis, which can be either minimized or maximized by this rule.

## Results

Al_2_ has five stationary states, 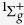, ^1^Π_*u*_, ^1^Δ_*g*_, ^3^Π_*u*_ and 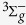, corresponding to the occupation of different molecular orbitals by two 3*p* electrons. Al_3_ has three stationary states, 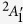, ^4^*A*_2_ and ^4^*B*_1_, corresponding to the occupation of different molecular orbitals by three 3*p* electrons. Their equilibrium nuclear geometries and corresponding total energies *E* are shown in Table 1. Hartree-Fock (HF) calculation predicts Al_2_ and Al_3_ to have ^3^Π_*u*_ and ^4^*A*_2_ high-spin ground states, respectively. Inclusion of Coulomb correlation by CAS-SCF (see Methods) maintains the high-spin ground state of Al_2_, but stabilizes the 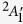 low-spin ground state of Al_3_. At the same time, high-spin terms of Al_2_ (^3^Π_*u*_, 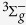) and Al_3_ (^4^*A*_2_, ^4^*B*_1_) become nearly degenerate; the energy difference between them is smaller than 0.01 a.u. The ^3^Π_*u*_ ground state for Al_2_ is consistent with experiment^34^, and our prediction of the 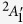 ground state for Al_3_ is corroborated by the Stern-Gerlach experiment^30^. More importantly, the ground state of Al_2_ is consistent with both Hund’s first and second rules, whereas Al_3_ violates both of them.

### Potential energy components

**Figure 1:**
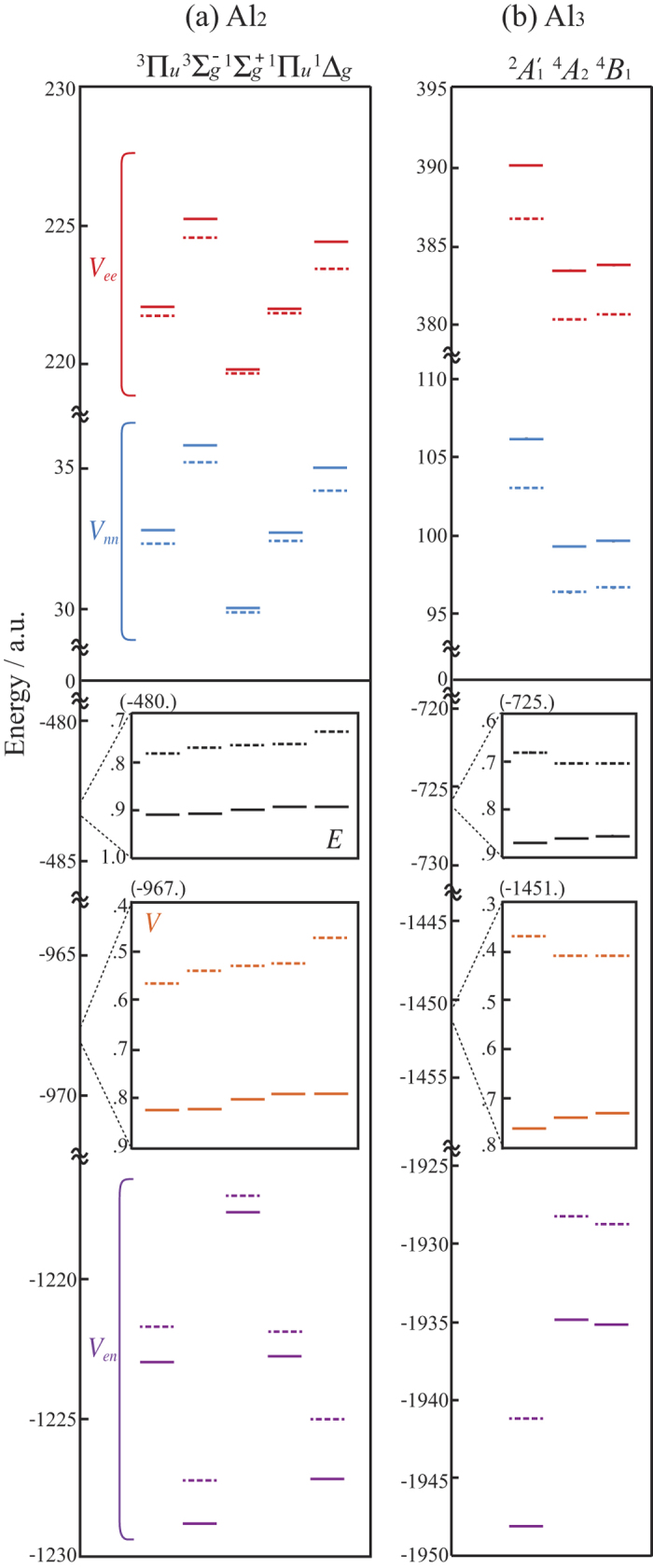
Energy components for each equilibrium state of Al_2_ and Al_3_. HF, CAS(6, 26) for Al_2_ and CAS(9, 18) for Al_3_ levels are dashed lines and solid lines, respectively. *E* (black), *V* (orange), *V*_*ee*_ (red), *V*_*nn*_ (blue), and *V*_*en*_ (purple) are given in hartree atomic units.

Traditionally, Hund’s rules have been interpreted as an energy gain due to the inter-electron repulsion potential energy *V*_*ee*_^35^,^36^,^37^, and more recently as an energy gain due to the electronuclear attraction *V*_*en*_^2^,^4^,^5^,^7^,^8^. In order to analyze whether or not similar energy lowering mechanisms can be invoked for Al_2_ and Al_3_, we decompose the total energies given in Table 1 into potential energy components shown in Fig. 1. In both HF (dashed lines) and CAS-SCF (solid lines) calculations for each stationary state of Al_2_ and Al_3_, repulsion terms *V*_*ee*_ (red lines) and *V*_*nn*_ (blue lines; inter-nuclear repulsion) are positive and the attraction term *V*_*en*_ (purple lines) is negative. The total energies *E* of Al_2_ and Al_3_ calculated by CAS-SCF always lie lower than those calculated by HF. For both Al_2_ and Al_3_, upon inclusion of Coulomb correlation by CAS(6, 26) and CAS(9, 18), respectively, the individual potential energy components *V*_*en*_, *V*_*ee*_ and *V*_*nn*_ composing *V* change as follows: both *V*_*ee*_ and *V*_*nn*_ increase and *V*_*en*_ decreases. The correlation energies *E*^*c*^ = *E*^*CAS*^ − *E*^*HF*^, along with *V*^*c*^, 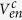, 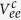, and 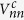, defined similarly, are unique to each molecular term; *E*^*c*^ < 0 always, and for the components we find 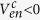, 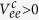, and 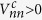.

For Al_2_, both HF and CAS-SCF predict


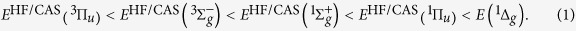


The correlation energies, however, exhibit the different trend


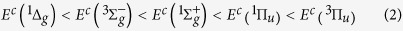


making the excitation energies smaller. For Al_3_, HF predicts


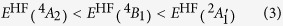


and CAS-SCF predicts


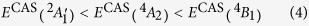


i.e. the level ordering is altered by correlation effects. For correlation energies we find


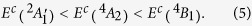


For both Al_2_ and Al_3_, the strongest correlation effect, i.e. greatest correlation energy *E*^*c*^, is observed for the ^1^Δ_*g*_ and 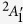 low-spin terms, respectively. For Al_3_, this correlation effect is strong enough to alter the level ordering of the molecular terms, but for Al_2_ not. Thus, the relative stability of the Al_2_ molecular terms can be discussed based on Fermi correlation (Pauli’s exclusion principle) and HF calculations, but for Al_3_, Coulomb correlation included by CAS-SCF is crucial for the description of molecular terms.

For Al_2_, Hund’s first and second rules predict


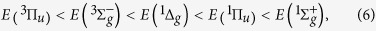


which is valid only for the spin-triplet terms. The spin-singlet terms exhibit an opposite trend to Hund’s second rule. Term stabilities have earlier been interpreted by either *V*_*ee*_^35^,^36^,^37^ or *V*_*en*_^2^,^4^,^5^,^7^,^8^, which imply that total energy differences are dominated by one potential energy component *V*_*i*_(*i* = *ee, en*, or *nn*), i.e., the total energy should follow the trend of this dominant *V*_*i*_. Figure 1, however, shows that


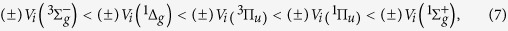


which is different to Eq. (1). Here the + sign corresponds to *i* = *en*, and the − sign to *i* = *ee* and *i* = *nn*. Clearly total energy trends do not follow any one particular potential energy component. Although the highest spin multiplicity (Hund’s first) rule does not follow any of the potential energy components *V*_*en*_, *V*_*ee*_, and *V*_*nn*_, the Ξ terms, when observed for spin-triplet and spin-singlet states individually, exhibit the following trends


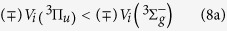



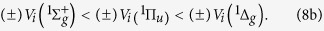


Note that Eq. (8a) has the opposite sign convention to Eqs (7) and (8b). Thus, for a given spin multiplicity, the potential energy components follow the same trend as total energies, but the sign may vary case by case!

### Fermi correlation and bond structure

Since clear term rules cannot be described based on the individual energy components discussed above, we turn our attention to the bond structures given in Table 2 for each molecular term. For Al_2_, inclusion of Coulomb correlation via CAS-SCF does not alter the relative stability of the Al_2_ terms, so the relative term stabilities can be understood purely based on Fermi correlation (Pauli exclusion principle). This leads to a simple description based on the bond structures of the different terms, i.e., the nodal structure of the wavefunction. Al_2_ has 3*pσ*_*g*_ and 3*pπ*_*u*_ bonding orbitals, and for the spin-singlet and spin-triplet terms, the most stable Ξ term has an occupied 3*pσ*_*g*_ orbital, i.e., the least node configuration. The stability of the spin-triplet ^3^Π_*u*_ against the spin-singlet 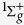 term also follows from HF theory. Starting from the nodeless 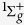 wavefunction, moving one electron from the 3*pσ*_*g*_ into a 3*pπ*_*u*_ with parallel spin (forming the ^3^Π_*u*_ term) lowers the total energy in three steps: (i) for fixed orbitals and Al–Al bond length, *V*_*ee*_ is lowered for spin parallel electrons^35^; (ii) relaxing the electronic orbitals lowers the total energy further; and (iii) relaxing the Al–Al bond length lowers the total energy further still. Repeating steps (ii) and (iii) obviously keeps lowering the total energy until convergence is found; these steps can be roughly associated to changes in *V*_*en*_ and *V*_*nn*_, respectively, but as seen in Fig. 1, for Al_2_ *V*_*ee*_ and *V*_*nn*_ actually increase despite the initial lowering of *V*_*ee*_ in step (i). For the Ξ terms we find that for a given spin multiplicity, the total energy increases as the number of nodes in the wavefunction increases.

### Coulomb correlation and bond structure

**Figure 2:**
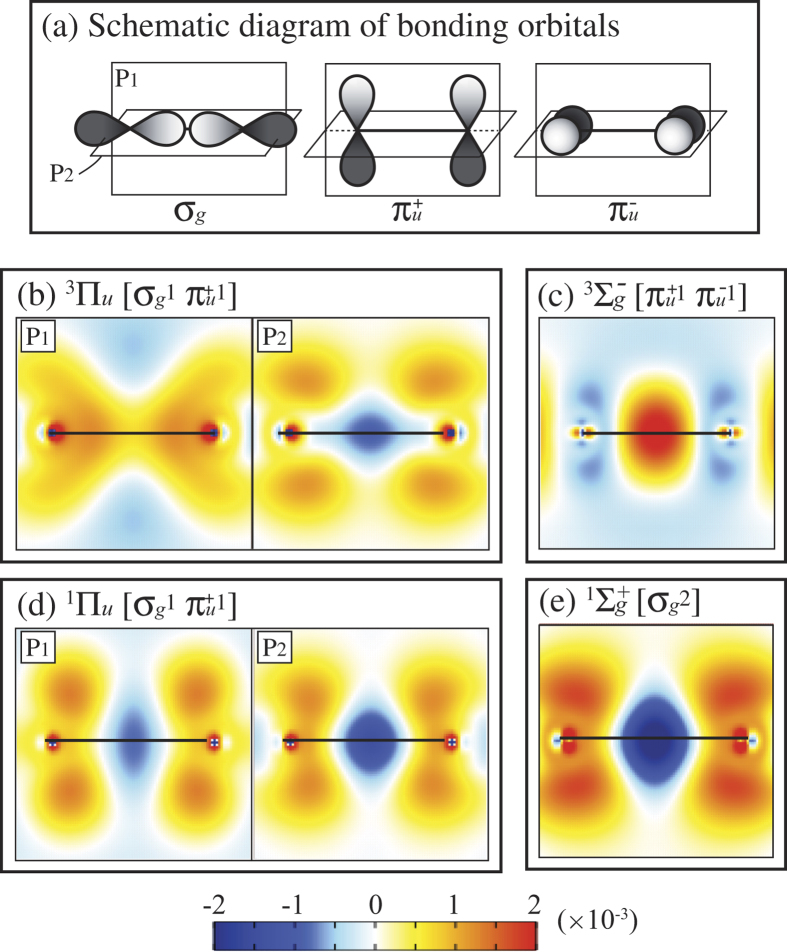
Schematic of bonding orbitals (**a**) and Coulomb correlation induced electron density distribution change *ρ*^*c*^ for stationary states of Al_2_ (**b–e**).Here *ρ*^*c*^ = *ρ*^CAS^ − *ρ*^HF^, where *ρ*^CAS^ is evaluated at CAS(6, 18).

**Figure 3:**
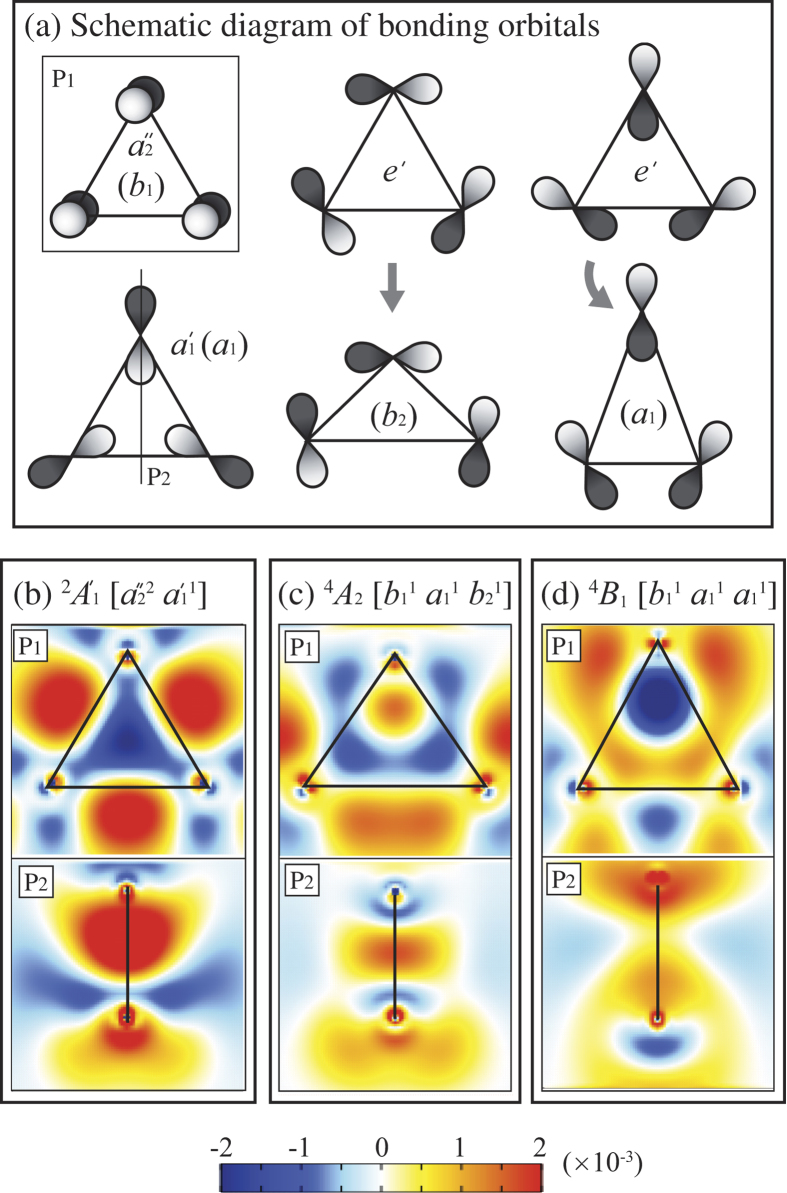
Schematic of bonding orbitals (**a**) and Coulomb correlation induced electron density distribution change *ρ*^*c*^ for stationary states of Al_3_ (**b–d**). Here *ρ*^CAS^ is evaluated at CAS(9, 12).

The above discussion fails for Al_3_. Inclusion of Coulomb correlation via CAS-SCF un-stabilizes the spin-quadruplet terms despite their possession of two electrons in 3*pσ* type orbitals (*a*_1_ and *b*_2_ for ^4^*A*_2_ and two *a*_1_s for ^4^*B*_1_) on the Al_3_ molecular plane. We analyze the effects of Coulomb correlation based on the electron density distribution change defined by *ρ*^*c*^ = *ρ*^CAS^ − *ρ*^HF^, where *ρ*^CAS^ and *ρ*^HF^ are the total electron densities calculated by CAS-SCF and HF, respectively. The crucial Coulomb correlation that alters the Al_3_ term stabilities occurs at CAS(9, 12), and therefore we evaluate *ρ*^CAS^ for Al_2_ and Al_3_ using CAS(6, 18) and CAS(9, 12), respectively. The *ρ*^*c*^ shown in Figs 2 and 3 for Al_2_ and Al_3_, respectively, are evaluated at the equilibrium nuclear configurations obtained by CAS(6, 18) and CAS(9, 12), respectively.

#### Al_2_

The Coulomb correlation effects are analyzed based on the bonding 3*pσ*_*g*_ and 3*pπ*_*u*_ orbitals shown in panel (a) of Fig. 2. Panels (b)–(e) of Fig. 2 show the electron density differences *ρ*^*c*^ for the ^3^Π_*u*_, 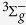, ^1^Π_*u*_, and 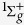 terms in the planes *P*_1_ and *P*_2_ corresponding to the 3*pσ*_*g*_ and 3*pπ*_*u*_ bonding orbitals. The blue areas indicate a depletion of electron density, and the yellow–orange–red areas an increase of electron density. For the 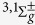 terms, these *P*_1_ and *P*_2_ planes are equivalent. The *ρ*^*c*^ analysis is omitted for the ^1^Δ_*g*_ term, which is not correctly represented in the HF calculation.

The CAS(6, 18) calculation includes various configurations including up to 3*d* orbitals, but the essence of the Coulomb correlation effects can be described based on the mixing of the bonding 3*pσ*_*g*_ and 3*pπ*_*u*_ orbitals. As shown in panels (b)–(e) of Fig. 2, the electron density distribution corresponding to orbitals occupied in HF theory (Table 2) is depleted, and increases corresponding to bonding orbitals not occupied in HF theory. For the ^3^Π_*u*_, ^1^Π_*u*_, and 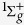 terms that in HF have an occupied 3*pσ*_*g*_ orbital, there is a depletion in *ρ*^*c*^ along the bond axis, and for the 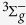 that in HF does not have an occupied 3*pσ*_*g*_ orbital, there is an increase. Likewise, *ρ*^*c*^ is negative in the regions corresponding to 3*pπ*_*u*_ orbitals occupied in HF theory, and positive in the regions where the 3*pπ*_*u*_ orbitals are not occupied in HF theory. Because all these Coulomb correlation effects essentially occur among the same set of orbitals, all of which are bonding, the effects are similar. Because the Coulomb correlation effects are similar for all terms, Coulomb correlation does not alter the relative stability of them, and the discussion above of term stability based on Fermi correlations and wavefunction nodal structure is sufficient.

#### Al_3_

Al_3_ has the 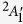 low-spin ground state, against expectations from Hund’s first rule or the spin-state stabilization mechanism for Al_2_ pertaining to HF theory. Thus, the energy lowering effect of Coulomb correlations is different for the low-spin 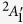 term and the high-spin ^4^*A*_2_ and ^4^*B*_1_ terms. The effect of these Coulomb correlations is discussed based on the 3*pσ* and 3*pπ* orbitals shown in panel (a) of Fig. 3. Panels (b)–(d) of Fig. 3 show the electron density differences *ρ*^*c*^ for the 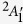, ^4^*A*_2_, and ^4^*B*_1_ terms in the plane *P*_1_ of the nuclei of Al_3_, and its perpendicular plane *P*_2_, which is a reflection symmetry plane of Al_3_. Notice that the nuclei of the spin-doublet 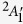 term form equilateral triangle, whereas the spin-quadruplet terms ^4^*A*_2_ and ^4^*B*_1_ correspond to isosceles triangles. Ensuingly, the bonding orbitals for the low-spin and high-spin terms are quite different.


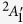
 has a doubly occupied 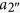 bonding orbital, a singly occupied 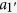 orbital, and a doubly degenerate *e*′ LUMO. The 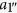 orbital is a *π* bond where the plane of nuclei is a nodal plane, and the *a*_1′_ is a *σ* bond with the charge density lobe in the center of the triangle. Both 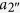 and 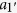 orbitals have *C*_3*v*_ symmetry, resulting in an equilateral trimer with *D*_3*h*_ symmetry. The doubly degenerate *e*′ LUMO corresponds to a *σ* bond with charge density lobes at all three sides of the triangle. The main Coulomb correlation effect is similar to what was discussed above for Al_2_. There is a depletion of electron in the regions corresponding to the 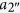 and 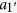 orbitals occupied in HF theory, and an increase in the region corresponding to the *e*′ orbitals, as seen in Fig. 3(b).

For the spin-quadruplet terms one of the 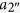 electrons occupies either one of the *e*′ orbitals. Individually these orbitals have the *C*_2*v*_ symmetry, yielding Jahn-Teller distorted isosceles triangles as described in Table 2. This changes the also symmetry species of the occupied 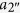 and 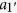 orbitals into *b*_1_ and *a*_1_, respectively, but these orbitals still maintain their nature as *π* and *σ* bonds with similar charge density lobes as described above for the equilateral triangle. The newly formed *a*_1_ or *b*_2_ orbitals for the ^4^*B*_1_ or ^4^*A*_2_ terms, have charge density lobes at the base or legs of the triangle, respectively, as shown in Fig. 3(a). The LUMO of the ^4^*B*_1_ and ^4^*A*_2_ terms are *b*_2_ and *a*_1_, respectively, i.e., the other one of the *e*′ orbitals for an equilateral triangle. For the spin-quadruplet terms, the main Coulomb correlation effect is the mixing of the *a*_1_ or *b*_2_ orbitals, which can be seen Fig. 3(c,d) as a depletion of electron density along the legs (base) of the triangle for ^4^*A*_2_ (^4^*B*_1_) and the corresponding along the base (legs) of the triangle.

Because of different symmetries, the Coulomb correlations for the low-spin and the high-spin terms of Al_3_ are fundamentally different. For the spin-doublet term, the main Coulomb correlation is the mixing of two occupied states and an unoccupied *doubly degenerate* state, whereas for the spin-quadruplet terms, the main Coulomb correlation is due to the mixing of one occupied and one unoccupied state. Coulomb correlation acts strongly among states nearby in energy and real space, and for the spin-quadruplet terms, the Jahn-Teller distortion imposes a severe limitation on the availability of such *nearby* states for mixing. This, combined with the fact that Coulomb correlation (even without geometrical distortions) is larger for low-spin configurations^2^ in total stabilizes the Al_3_ low-spin ground state. Thus, both Hund’s first rule and the mechanism that stabilizes the high-spin ground state of Al_2_ are violated because the breaking of symmetry of the Al_3_ spin-quadruplet configurations reduces their Coulomb correlation. Note that Hund’s maximum spin multiplicity rule is violated under exactly the opposite conditions as postulated by Kutzelnigg and Morgan^20^.

### Larger clusters

Application of the term rules described above for other clusters is straight forward. We illustrate this generalization by predicting ground state terms for Al_4_ and Al_5_. For both clusters, we consider previously described planar and pyramidal structures^26^,^28^,^38^; incidentally, our discussion below offers a new interpretation for why planar geometries are favored against pyramidal ones^39^. We predict ^3^*B*_1*u*_ and ^2^*B*_1_ ground states for Al_4_ and Al_5_, respectively, well in agreement with previous works^21^,^26^,^28^. The structures and spin multiplicities agree also with density-functional calculations^29^,^38^, which however give no information of the symmetry species Ξ.

#### Al_4_

Al_4_ can have spin-singlet, spin-triplet, and spin-quintet states due to different configurations of four 3*p* electrons, shown in Table 3. The HOMO of any of the Al_4_ terms with pyramidal structure (3-fold degenerate 2*t*_1_ orbitals) do not form *σ*-type bonds, so for any spin multiplicity, the least node wavefunctions corresponds to a planar geometry. The planar Al_4_ spin-quintet terms (high spin) always have at least one occupied antibonding molecular orbital, such as 2*b*_3*u*_, 2*b*_2*u*_, 2*b*_3*g*_, and 2*b*_2*g*_, whereas the spin-triplet and singlet terms ^3^*B*_1*u*_, ^3^*A*_*u*_, ^3^*B*_1*g*_, and ^1^*A*_*g*_ have valence electrons occupying in bonding orbitals (1*b*_1*g*_, 1*b*_1*u*_, and 3*a*_*g*_). Thus, Fermi correlation stabilizes the spin-triplet terms with possession of most-occupied *σ*-type bonding orbitals, i.e., ^3^*B*_1*u*_ state. Coulomb correlation, which enhances the electron density on the nodal plane of HOMO(s), makes Al-Al bonds on the molecular plane stronger for both ^3^*B*_1*u*_ and ^1^*A*_*g*_ states. As seen in stability of Al_2_’s spin triplet terms, such Coulomb correlation effect cannot reverse the relative stability for ^3^*B*_1*u*_ and ^1^*A*_*g*_, and thus we predict ^3^*B*_1*u*_ as the ground state term of Al_4_.

#### Al_5_

Al_5_ can have spin multiplicities up to spin-sextet due to different configurations of five 3*p*-electrons. All pyramidal, and the planar spin-sextet terms have at least one electron in antibonding or nonbonding orbitals (see Table 4), and thus cannot be more stable than the planar spin-doublet or spin-quadruplet terms. The planar spin-quadruplet terms (intermediate spin state) only have partial bonds, such as 3*b*_1_, 4*b*_1_, and 2*b*_2_, which leaves only spin-doublet terms with strong *σ*-type bonds. Thus for Al_5_, we predict the planar ^2^*B*_1_ spin-doublet term, which has the least node structure ground state.

## Discussion

Our benchmark first principles calculation predicts the ^3^Π_*u*_ (high-spin) and 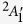 (low-spin) ground states for Al_2_ and Al_3_, respectively. Detailed analysis of potential energy components of the total energy reveal that previous interpretations, attributing atomic or molecular term stabilization to either *V*_*ee*_^35^,^36^ or *V*_*en*_^2^,^5^,^8^ are, in general, not valid for multi-atom systems. The relative stability of the Ξ terms for a given spin multiplicity for either Al_2_ and Al_3_ follows simple arguments based on bonding structures: For a given spin multiplicity the Ξ term possessing the most-occupied *σ* bonding orbitals (least node structure) is stabilized within the one-electron orbital picture according to Hartree-Fock (HF) theory. In addition, HF theory tends to stabilize the high-spin term due to Fermi correlation (Pauli exclusion principle). Coulomb correlation lowers the energy by mixing some of the orbitals occupied in HF theory with nearby unoccupied orbitals. For Al_2_, the Coulomb correlation effects are similar for all terms, but for Al_3_, Coulomb correlation alters the relative term stability. For Al_3_, breaking of symmetry of the the spin-quadruplet terms significantly limits the orbital mixing and energy lowering by Coulomb correlation. The high symmetry of the spin-doublet term, on the other hand, allows for mixing with *degenerate* levels followed by a much larger energy lowering by Coulomb correlation, stabilizing the low-spin 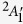 ground state of Al_3_. These stabilization mechanisms are not specific for Al clusters, and serve as simple term rules to determine the ground state of arbitrary multi-atomic systems. We demonstrate this predictive power by predicting ^3^*B*_1*u*_ and ^2^*B*_1_ ground states for Al_4_ and Al_5_, respectively.

## Methods

The total energy *E* of the ^2*S*+1^Ξ*g*/*u* term of an Al_*n*_ cluster in the Born-Oppenheimer approximation is given by 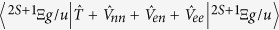, where 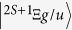 is a many-electron wavefunction and the operators 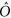 give the electron kinetic energy, the inter-nuclear repulsion, the electronuclear attraction, and the inter-electron repulsion, respectively. The expectation values 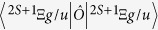 for each operator 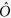, henceforth denoted as *O*(^2*S*+1^Ξ*g*/*u*), are calculated using the GAMESS package^40^. We use both Hartree-Fock (HF) method and complete active space self-consistent field (CAS-SCF) method. Our CAS-SCF many-electron wavefunctions contain configuration interactions among the 3*s* and 3*p* valence shells and empty 3*d*-derived orbitals: CAS(6, 26) and CAS(9, 18) for Al_2_ and Al_3_, respectively. CAS(n, m) stands for a CAS-SCF calculation with *n* active spaces and *m* active electrons. Atomic orbitals are expanded within the aug-cc-pVTZ basis set, and all nuclear positions are relaxed. This gives a virial ratio of −*V*/*T* = 2.00000 ± 0.00003 for each molecular term ^2*S*+1^Ξ*g*/*u*.

## Additional Information

**How to cite this article**: Yoshida, D. and Raebiger, H. Term rules for simple metal clusters. *Sci. Rep.* **5**, 15760; doi: 10.1038/srep15760 (2015).

## Figures and Tables

**Table 1:** Total energies and equilibrium structures of Al_2_ and Al_3_.

Al_2_	HF		CAS(6, 18)		CAS(6, 26)				
	*E*	*r*	*E*	*r*	*E*	*r*			
^3^Π_*u*_	−483.7824(0)	2.770	−483.8939(23)	2.714	−483.9082(13)	2.729			
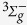	−483.7700(2)	2.544	−483.8892(27)	2.474	−483.9064(16)	2.501			
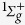	−483.7647(1)	2.996	−483.8830(21)	2.972	−483.8980(12)	2.980			
^1^Π_*u*_	−483.7627(1)^a^	2.754	−483.8775(14)	2.745	−483.8925(8)	2.749			
^1^Δ_*g*_	−483.7369(6)	2.619	−483.8760(28)	2.528	−483.8915(15)	2.556			
Al_3_	HF			CAS(9, 12)			CAS(9, 18)		
	*E*	*r*	*θ*	*E*	*r*	*θ*	*E*	*r*	*θ*
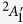	−725.6780(30)	2.607	60.0°	−725.8100(74)	2.567	60.0°	−725.8655(73)	2.530	60.0°
^4^*A*_2_	−725.7007(27)	2.651	71.7°	−725.7962(63)	2.620	69.6°	−725.8554(64)	2.591	70.0°
^4^*B*_1_	−725.6995(25)	2.868	54.2°	−725.7969(63)	2.784	55.8°	−725.8496(67)	2.760	55.6°

Total energies *E* are given in hartree atomic unit (a.u), and Al-Al bond *r* lengths in Å. For Al_3_ *r* is the length of either leg of an isosceles triangle with vertex angle *θ*. Values in parenthesis are maximum errors within the virial theorem.

^a^CAS(2, 2) calculation using doubly degenerate configurations in 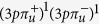.

**Table 2:** Electronic configurations of valence electrons of Al_2_ and Al_3_.

Al_2_		Al_3_	
^3^Π_*u*_	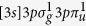	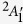	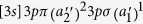
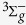	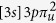	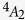	[3*s*]3*pπ*(*b*1)^1^3*pσ*(*a*1)^1^3*pσ*(*b*2)^1^
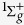	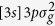	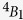	[3*s*]3*pπ*(*b*1)^1^3*pσ*(*a*1)^1^3*pσ*(*a*1)^1^
^1^Π_*u*_	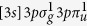		
^1^Δ_*g*_	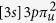		

[3*s*] represents the configurations of 3*s* electrons; In Al_2_, 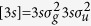, In Al_3_, 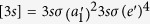 for 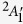, and [3*s*] = 3*sσ*(*a*_1_)^2^3*sσ*(*a*_1_)^2^3*sσ*(*b*_2_)^2^ for ^4^*A*_2_ and ^4^*B*_1_.

**Table 3:**
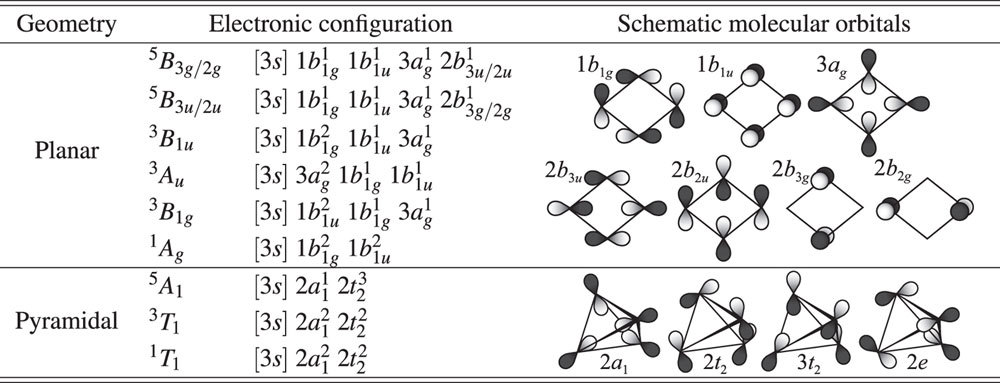
Electronic configurations of valence electrons for planar and pyramidal geometries for Al_4_.

Here 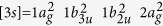 for the planar, and 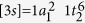 for the pyramidal geometries.

**Table 4:**
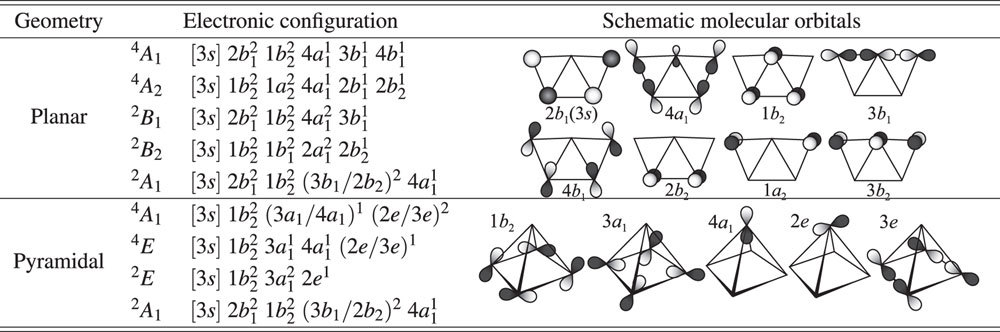
Electronic configurations of valence electrons for planar and pyramidal geometries for Al_5_.

Here 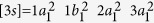 for the planar, and 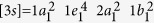 for the pyramidal geometries.

## References

[b1] Hund F. Zur Deutung verwickelter Spektren, insbesondere der Elemente Scandium bis Nickel. Z. Physik 33, 345–371 (1925).

[b2] Katriel J. & Pauncz R. Theoretical interpretation of Hund’s rule. Adv. in Quantum Chem. 10, 143–185 (1977).

[b3] Boyd R. J. A quantum mechanical explanation for Hund’s multiplicity rule. Nature 310, 480–481 (1984).

[b4] Darvesh K. V., Fricker P. D. & Boyd R. J. Interpretation of Hund’s rule for first-row hydrides AH (A = Li, B, N, F). J. Chem. Phys. 94, 3480–3484 (1990).

[b5] Oyamada T., Hongo K., Kawazoe Y. & Yasuhara H. Unified interpretation of Hund’s first and second rules for 2*p* and 3*p* atoms. J. Chem. Phys. 133, 164113 (2010). 21033781 10.1063/1.3488099

[b6] Tanaka K., Nomura T. & Noro T. Ab initio SCF CI calculations on the ground and π-π* excited states of the pyrrole molecule and its positive ion. J. Chem. Phys. 67, 5738–3741 (1977).

[b7] Darvesh K. V. & Boyd R. J. Hund’s rule and singlet-triplet energy differences for the lowest *nπ** states of formaldehyde, H_2_CO. J. Chem. Phys. 90, 5638–5643 (1989).

[b8] Maruyama Y., Hongo K., Tachikawa M., Kawazoe Y. & Yasuhara H. Ab initio interpretation of Hund’s rule for the methylene molecule: Variational optimization of its molecular geometries and energy component analysis. Int J Quantum Chem 108, 731–743 (2008).

[b9] Slipchenko *et al.* 5-Dehydro-1,3-quinodimethane: A Hydrocarbon with an Open-Shell Doublet Ground State. Angew Chem Int Edit 43, 742–745 (2004). 10.1002/anie.200352990 14755709

[b10] Fumi F. G. & Parr R. G. Electronic States of Diatomic Molecules: The Oxygen Molecule. J. Chem. Phys. 21, 1864 (1953).

[b11] Padgett A. A. & Griffing V. LCAO-MO SCF Study of B2. J. Chem. Phys. 30, 1286 (1959).

[b12] Bender C. F. & Davidson E. R. Electronic Structure of the B2 Molecule. J. Chem. Phys. 46, 3313–3319 (1967).

[b13] YoshimineM. The second 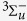 state of O_2_. J. Chem. Phys. 64, 2254 (1976).

[b14] StevensW. J. & KraussM. The electronic structure of the ground and excited states of 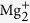 and Mg_2_. J. Chem. Phys. 67, 1977 (1977).

[b15] TatewakiH. *et al.* Configuration-Interaction study of lower excited states of O_2_: Valence and Rydberg characters of the two lowest 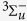 states. Int. J. Quantum Chem. 15, 533–545 (1979).

[b16] Delyagina I. A., Kokh D. B. & Pravilov A. M. Study of the covalent and triplet ionic-pairing states of the fluorine molecule with the MRDCI method. Optics and Spectroscopy 94, 170–178 (2003).

[b17] Bytautas L., Matsunaga N., Nagata T., Gordon M. S. & Ruedenberg K. Accurate ab initio potential energy curve of F_2_. II. Core-valence correlations, relativistic contributions, and long-range interactions. J. Chem. Phys. 127, 204301 (2007). 18052421 10.1063/1.2801989

[b18] Su P. *et al.* Bonding Conundrums in the C_2_ Molecule: A Valence Bond Study. J. Chem. Theory Comput. 7, 121–130 (2011). 10.1021/ct100577v 26606225

[b19] Magoulas I., Kalemos A. & Mavridis A. An ab initio study of the electronic structure of BF and BF^+^. J. Chem. Phys. 138, 104312 (2013). 23514494 10.1063/1.4793738

[b20] Kutzelnigg W. & Morgan J. D. III. Hund’s rules. Z Phys D 36, 197–214 (1996).

[b21] Pacchioni G., Plavšić D. & Koutecký J. Chemical bonding and electronic structure of small homonuclear clusters of elements of groups IA, IIA, IIIA and IVA. Ber. Bunsenges. Phys. Chem. 87, 503–512 (1983).

[b22] Pacchioni G. & Koutecký J. Silicon and germanium clusters. A theoretical study of their electronic structures and properties. J. Chem. Phys. 84, 3301–3310 (1986).

[b23] Pacchioni G. & Koutecký J. Ab initio MRD CI investigation of the optical spectra of C_4_ and C_5_ clusters. J. Chem. Phys. 88, 1066–1073 (1988).

[b24] Zabala N., Puska M. J. & Nieminen R. M. Spontaneous magnetization of simple metal nanowires. Phys. Rev. Lett. 80, 3336–3339 (1998).

[b25] Ayuela A., Raebiger H., Puska M. & Nieminen R. Spontaneous magnetization of aluminum nanowires deposited on the NaCl(100) surface. Phys. Rev. B 66, 035417 (2002).

[b26] Pettersson L., Bauschlicher C. W. & Halicioglu T. Small Al clusters. II. Structure and binding in Al_*n*_ (*n* = 2-6, 13). J. Chem. Phys. 87, 2205–2213 (1987).

[b27] Bauschlicher C. W., Partridge H., Langhoff S. R., Taylor P. R. & Walch S. P. Accurate ab initio calculations which demonstrate a ^3^Π_*u*_ ground state for Al_2_. J. Chem. Phys. 86, 7007–7012 (1987).

[b28] Meier U., Peyerimhoff S. D. & Grein F. Ab initio MRD-CI study of neutral and charged Ga_2_, Ga_3_ and Ga_4_ clusters and comparison with corresponding boron and aluminum clusters. Z. Physik D Atom. Mol. Cl. 17, 209–224 (1990).

[b29] Rao B. K. & Jena P. Evolution of the electronic structure and properties of neutral and charged aluminum clusters: A comprehensive analysis. J. Chem. Phys. 111, 1890–1904 (1999).

[b30] Cox D. M., Trevor D. J., Whetten R. L., Rohlfing E. A. & Kaldor A. Aluminum clusters: Magnetic properties. J. Chem. Phys. 84, 4651–4656 (1986).

[b31] Howard J. A., Sutcliffe R., Tse J. S., Dahmane H. & Mile B. Electron spin resonance spectra of the aluminum trimer in hydrocarbon matrices: A quartet ^4^A_2_ state. J. Phys. Chem. 89, 3595–3599 (1985).

[b32] Tse J. S. Stability and potential energy surface of the three low lying electronic states of Al_3_. J. Chem. Phys. 92, 2488–2494 (1990).

[b33] Hamrick Y. M., Vanzee R. J. & Weltner W. Electron-spin resonance and ground states of the boron and aluminum trimers. J. Chem. Phys. 96, 1767–1775 (1992).

[b34] Cai M. F., Dzugan T. P. & Bondybey V. E. Fluorescene studies of laser vaporized aluminum: Evidence for a ^3^Π_*u*_ ground state of aluminum dimer. Chemical Physics Letters 155, 430–436 (1989).

[b35] Slater J. C. The theory of complex spectra. Phys. Rev. 34, 1293–1322 (1929).

[b36] Van Vleck J. H. Valence strength and the magnetism of complex salts. J. Chem. Phys. 3, 807 (1935).

[b37] Colpa J. P. & Brown R. E. The inequality formulation of Hund’s rule and a reinterpretation of singlet–triplet energy differences, generalized for molecules at equilibrium geometry. J. Chem. Phys. 68, 4248–4251 (1978).

[b38] Jones R. O. Structure and bonding in small aluminum clusters. Phys. Rev. Lett. 67, 224 (1991). 10044526 10.1103/PhysRevLett.67.224

[b39] GeskeG. D., BoldyrevA. I., LiX. & WangL.-S. On the origin of planarity in 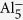 and Al_5_ clusters: The importance of a four-center peripheral bond. J. Chem. Phys. 113, 5130 (2000).

[b40] Schmidt M. W. *et al.* General atomic and molecular electronic structure system. J. Comput. Chem. 14, 1347–1363 (1993).

