# Erratum: Term rules for simple metal clusters

**DOI:** 10.1038/srep17982

**Published:** 2016-01-12

**Authors:** Daisuke Yoshida, Hannes Raebiger

Scientific Reports 5: Article number: 1576010.1038/srep15760; published online 10262015; updated on 01122016

In the Methods section of this Article, all instances of ‘g/u’ should have been removed. The Methods section should read:

The total energy *E* of the ^2*S*+1^Ξ term of an Al_*n*_ cluster in Born-Oppenheimer approximation is given by 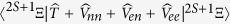, where 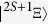 is a many-electron wavefunction and the operators 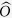 give the electron kinetic energy, the inter-nuclear repulsion, the electronuclear attraction, and the inter-electron respectively. The expectation values 
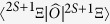
 for each operator 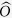, henceforth denotes as *O*(^2*S*+1^Ξ), are calculated using the GAMESS package.^40^ We use both Hartree-Fock (HF) method and complete active space self-consistent field (CAS-SCF) method. Our CAC-SCF many-evectron wavefunctions contain configuration interactions among the 3*s* and 3*p* valence shell and empty 3*d*-derived orbitals: CAS(6,26) and CAS(9,18) for Al_2_ and Al_3_, respectively. CAS(n,m) stands for a CAS-SCF calculation with *n* active spaces and *m* active electrons. Atomic orbitals are expanded within the aur-cc-pVTZ basis set, and all nuclear positions are relaxed. This gives a virial ratio of –*V*/*T* *=* 2.00000 ± 0.00003 for each molecular term ^2*S*+1^Ξ.

